# Possible associations between callers’ degree-of-worry and their socioeconomic status when contacting out-of-hours services: a prospective cohort study

**DOI:** 10.1186/s12873-021-00452-0

**Published:** 2021-04-28

**Authors:** Sita LeBlanc Thilsted, Fredrik Folke, Janne S. Tolstrup, Lau Caspar Thygesen, Hejdi Gamst-Jensen

**Affiliations:** 1 Copenhagen Emergency Medical Services, University of Copenhagen, Copenhagen, Denmark; 2 National Institute of Public Health, University of Southern Denmark, Odense, Denmark; 3 Clinical Research Centre, Copenhagen University Hospital Hvidovre, Hvidovre, Denmark

**Keywords:** Decision support systems, Emergency medicine, Telephone hotlines, Triage, Patient-centred care, Decision making, Help-seeking behaviour, Socioeconomic status, Marital status, Ethnicity

## Abstract

**Background:**

Telephone triage within out-of-hours (OOH) services aims to ascertain the urgency of a caller’s medical condition in order to determine the correct type of health care needed, ensuring patient safety. To improve the triage process by increasing p*atient-centred communication*, a triage tool has been developed, whereby callers are asked to rate their degree-of-worry (DOW) as a measure of self-evaluated urgency. Studies show that low socioeconomic status (SES), being single and non-Western ethnicity are associated to low self-rated health and high morbidity and these factors may also be associated with high DOW. The aim of this paper was to examine if low SES, being single and non-Western ethnicity were associated to high DOW of callers contacting OOH services.

**Methods:**

A prospective cohort study design, at the OOH services for the Capital Region of Denmark. Over 2 weeks, 6869 of 38,787 callers met the inclusion criteria: ≥18 years, patients themselves or close relative/friend, reported DOW, had a valid personal identification number and gave informed consent. Callers were asked to report their DOW (1 = minimal worry to 5 = maximal worry), which was dichotomized into low (1–3) and high (4, 5) DOW and linked to data from electronical medical records and Statistics Denmark. Socioeconomic factors (education and annual household income), marital status and ethnicity were assessed in relation to DOW by logistic regression.

**Results:**

High DOW was reported by 38.2% of the participants. Low SES (low educational level; OR 1.5, 95% CI 1.3–1.7 and low annual household income; 1.5, 1.3–1.6) was associated with high DOW and so too was being single; 1.2, 1.1–1.3 and of non-Western ethnicity; 2.9, 2.5–3.4.

**Conclusions:**

Knowledge of the association of low SES, marital status as single and non-Western ethnicity with high DOW among callers to OOH services may give call handlers a better understanding of callers’ DOW. If this does not correspond to the call handler’s perception of urgency, this knowledge may further encourage patient-centred communication, aid the triage process and increase patient safety. A better understanding of socioeconomic variables and their relation to callers’ DOW gives direction for future research to improve telephone triage of OOH services.

## Background

Prehospital telephone triage within out-of-hours (OOH) services is recognised as a means to reduce pressure and overcrowding of emergency departments and OOH clinics [1]. Telephone triage aims to ascertain the urgency of a caller’s medical condition in order to determine the correct type of health care needed, thus ensuring patient safety. However, due to the lack of non-verbal cues, urgency assessment is more challenging than in face-to-face consultations [2]. Triage tools, e.g. computerised decision support systems are therefore used to aid the triage process [3]. However, these tools focus mainly on medical information and less on affective information [4].

Patient-centered communication is defined as eliciting and understanding the patient’s perspective of the illness including their wants, needs and preferences and acknowledging the patient’s psychosocial and cultural circumstances, so a shared treatment goal can be reached [5]. It is now identified as a central aspect for patient safety and is key to improving the quality of health care [6, 7]. It has been suggested that patients expressing a potential need for hospitalisation should be thoroughly examined for possible severe illness [8] and that patients’ verbalisation of concerns and needs should be viewed as valid and encouraged by health care professionals [9, 10]. This also pertains to the telephone triage process within OOH services. Therefore, a triage tool has been developed, whereby callers are asked to rate their degree-of-worry (DOW), on a five-point scale, as a measure of self-evaluated urgency [11]. The goal is to increase p*atient-centred communication* by encouraging patients’ participation and thereby aiding call handlers in determining urgency and type of health care needed, potentially increasing patient safety. DOW has previously been shown to be feasible and associated with callers’ illness representation [12]. It has also been shown that high DOW is strongly associated with hospitalisation [13], but that call handlers’ awareness of callers’ DOW had no effect on triage response [14]. There continues to be many unexamined aspects regarding the implementation of DOW as a patient-reported outcome measure.

There is ample evidence that socioeconomic circumstances, e.g. education, household income and marital status influence individuals’ health outcomes [15, 16] and that individuals with low socioeconomic status (SES) are more likely to have low self-reported health [17] and high morbidity and mortality [18]. Furthermore, it has been demonstrated that individuals with low SES more often consult emergency departments and OOH services, even when their health status is taken into account [19, 20]. This is assumed partly due to low health literacy [21], leading to difficulties in navigating the health care system, whereby individuals contact OOH services as an alternative to their general practitioner (GP) [22]. Evidence also suggest that marital status affects individuals health, where married individuals typically lead longer and healthier lives than singles [23]. Finally, ethnicity is an important factor for health status and health-care utilization [24], where non-Western Danes report a lower self-rated health and have higher rates of morbidity compared to ethnic Danes [25].

Knowing that individuals with low SES, single marital status and non-Western ethnicity are generally more ill, one could hypothesise that when calling OOH services these individuals would report a higher DOW. The association of callers’ SES status, marital status and ethnicity with their DOW is, however, unknown. A greater insight in these possible associations may give call handlers a better understanding of callers’ DOW reported during telephone triage. An increased knowledge and understanding of this triage tool may further encourage patient-centred communication, aid the assessment of urgency of a caller’s medical condition and increase patient safety. Furthermore, a better understanding of the possible association of callers’ SES, marital status and ethnicity with DOW when contacting OOH services may add to the growing awareness of the socioeconomic inequality in health and healthcare and the continuing necessity of minimizing this gap.

## Method

### Aim

The aim of this paper is to examine if low SES, marital status and non-Western ethnicity are associated to high DOW of callers contacting OOH services.

### Study design

A prospective cohort study. Data collected was linked with data on individual SES from Statistics Denmark.

### Setting

The Copenhagen Emergency Medical Services, the Capital Region of Denmark and the OOH services are integrated into one organisation and can be reached through two telephone numbers; 112, the Copenhagen Emergency Medical Services for life-threatening emergencies and 1813, the OOH services for acute, non-emergent medical calls. The OOH services are available from 4 pm to 8 am on weekdays and around the clock on weekends and holidays. Individuals may also call the OOH services for a referral to an emergency department, if they cannot get in touch with their GP during regular working hours. All access to acute care is pre-assessed by telephone triage. Annually, approximately one million calls are handled by call handlers (nurses/physicians) who triage the caller to self-care, GP, home visit, face-to-face assessment/consultation at a hospital, or direct hospitalisation [26, 27]. Call handlers use a criterion-based electronic triage tool, which is locally developed and not validated.

### Data collection

**Fig. 1:**
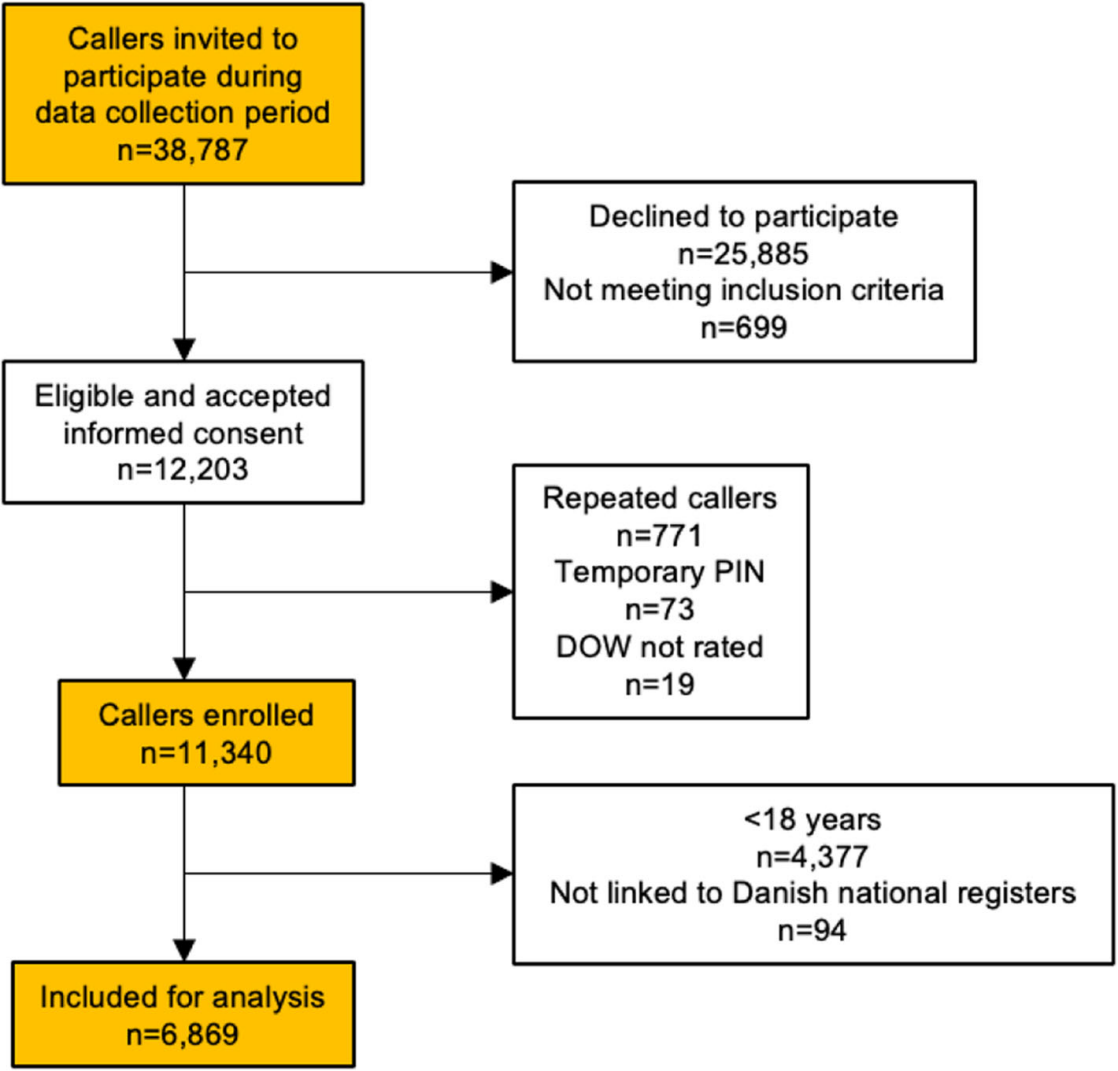
Flow diagram of participants’ inclusion and exclusion criteria

Callers to the OOH services from January 24th to February 9th, 2017, were invited to participate in the study. Inclusion criteria were age ≥ 18 years of the patient and being able to link patient’s unique PIN (personal identification) number to Statistics Denmark. Exclusion criteria were lack of consent, not being the patient themselves or a close relative/friend to the patient, repeat callers, not having a permanent PIN and not rating DOW (see Fig. 1). Via telephone survey, participants electronically reported their DOW on a five-point scale (1 = minimal worry to 5 = maximum worry) before being put through to a call handler. Callers’ DOW was stored in their electronic medical record. An in-depth description of this data collection has been published previously [13].

### Data sources

Data was derived from two different sources:

1. At the OOH services, callers’ medical information, including PIN, gender and age are registered in their electronic medical record. Data on DOW was also stored in callers’ electronic medical record.
2. Statistics Denmark is the central data resource for individual level data on e.g. highest attained education, annual household income, marital status and country of origin, which is linkable to each resident’s unique PIN [28].

### Variables

The socioeconomic variables were categorized as follows:

1. educational level; low: early childhood, primary and lower secondary education; International Standard Classification of Education (ISCED) = 0–2, middle: upper and post-secondary education; ISCED = 3–4 and high: tertiary education, bachelor’s, master’s and doctoral level; ISCED = 5–8.
2. annual household income; low: Danish Kroner (DKK) < 189,460, middle: DKK 189,460–294,180 and high: DKK > 294,180,
3. marital status; single or married/cohabiting
4. ethnicity; Danish, non-Danish Western and non-Western

Age was categorized by 18–30, 31–40, 41–50, 51–60, 61–70, 71–80 and > 80 years.

### Outcome measure

For statistical purposes, DOW was dichotomized into two groups: low (DOW = 1–3) and high (DOW = 4–5), based on the association between DOW and hospitalization, where DOW = 4–5 showed significantly higher odds for hospitalization than DOW = 1–3 [13].

### Analysis

Associations between education, annual household income, marital status and ethnicity and high DOW were analysed separately, using logistic regression models adjusted for gender and continuous age. Results were reported as odds ratios (OR) and 95% confidence intervals (95% CI).

## Results

### Participants

A total of 38,787 callers were invited to participate in the study. Of these, 25,885 callers declined participation, 699 were not patients themselves or a close relative/friend of the patient, 771 were repeated callers, 73 had a temporary PIN [29] and 19 did not rate their DOW. This resulted in inclusion of 11,340 callers, where only adult callers (≥18 years) (*n* = 4377 excluded) that could be linked by their unique PIN to registers at Statistics Denmark (*n* = 94 excluded), were included in the present study, resulting in analysis of a total of 6869 callers (see Fig. 1).

DOW was rated as high by 38.2% of the 6869 callers. The study population consisted mainly of females (58.3%), with the proportions of females being similar in both the low (57.9%) and high (59.0%) DOW category. The mean age of those reporting a high DOW was 51.6 (standard deviation (SD) 20.9) years compared to 43.4 (SD 18.7) years for low DOW. The proportion of callers reporting high DOW increased with age; with 29.5% of callers aged 18–30 years reporting high DOW compared to 57.5% of callers aged > 80 years.

Educational level was registered for 6573 callers, with most callers (2820; 41.1%) having a middle educational level. Information on household income was available for 6129 callers, with the mean annual household income (DKK 266,986 (SD 209,711)) falling in the middle category. The majority of the callers (3847; 56%) was married or cohabiting. Finally, the majority (83.1%) of callers were of Danish ethnicity, whereas only 4.2% were of non-Danish Western ethnicity and 12.7% were of non-Western ethnicity (Table 1).

**Table 1:** Descriptive information of study participants (n, %)

Demographics and socioeconomic variables on Degree-of-worry (DOW)			
	Total *n* = 6869 (%)	Low (1–3) *n* = 4243 (61.8)	High (4–5) *n* = 2626 (38.2)
Gender			
Female	4007	2456 (61.3)	1551 (38.7)
Male	2862	1787 (62.4)	1075 (37.6)
Age (year)			
Mean, SD	46.5 ± 20.0	43.4 ± 18.7	51.6 ± 20.9
18–30	1920	1354 (70.5)	566 (29.5)
31–40	1175	836 (71.1)	339 (28.9)
41–50	1072	681 (63.5)	391 (36.5)
51–60	915	527 (57,6)	388 (42.4)
61–70	694	360 (51,9)	334 (48.1)
71–80	639	292 (45.7)	347 (54.3)
> 80	454	193 (42.5)	261 (57.5)
Educational level			
Low	1763	968 (54.9)	795 (45.1)
Middle	2820	1773 (62.9)	1047 (37.1)
High	1990	1349 (67.8)	641 (32.2)
Missing	296	153 (51.7)	143 (48.3)
Household income, DKK			
Low (< 189,460)	2211	1218 (55.1)	993 (44.9)
Middle (189,460-291,175)	1978	1225 (61.9)	753 (38.1)
High (> 291,175)	1940	1300 (67.0)	640 (33.0)
Missing	740	500 (67.6)	240 (32.4)
Marital status			
Married/cohabiting	3847	2450 (63.7)	1397 (36.3)
Single	3022	1793 (59.3)	1229 (40.7)
Ethnicity			
Danish	5710	3678 (64.4)	2032 (35.6)
Non-Danish Western	287	170 (59.2)	117 (40.8)
Non-Western	872	395 (45.3)	477 (54.7)

### Associations between socioeconomic variables and DOW

Low educational level was associated with high DOW (OR 1.5, 95% CI 1.3 to 1.7), compared to middle educational level. Whereas, a high educational level was associated to reporting a low DOW compared to middle educational level. Low annual household income was associated with high DOW (OR 1.4, 95% CI 1.2 to 1.6), compared to middle annual household income. Whereas high annual household income was associated to a low DOW compared to middle annual household income. Being single was associated with high DOW (OR 1.3, 95% CI 1.1 to 1.5) compared to being married or cohabiting. Non-Western ethnicity was associated with high DOW (OR 2.9, 95% CI 2.5 to 3.4), compared to Danish ethnicity, but there was no significant association with non-Danish Western ethnicity (see Table 2).

**Table 2:** Odds ratio (OR, 95% CI) for reporting high degree-of-worry (DOW) by socioeconomic status adjusted for gender and age

	OR (95% CI)
Educational level	
Low	1.5 (1.3 to 1.7)
Middle	1.0 (Reference)
High	0.8 (0.7 to 0.9)
Missing	1.7 (1.3 to 2.2)
Household income (DKK)	
Low	1.4 (1.2 to 1.6)
Middle	1.0 (Reference)
Upper	0.8 (0.7 to 0.9)
Missing	1.1 (0.9 to 1.3)
Marital Status	
Married/cohabiting	1.0 (Reference)
Single	1.3 (1.1 to 1.5)
Ethnicity	
Danish	1.0 (Reference)
Non-Danish Western	1.3 (1.0 to 1.6)
Non-Western	2.9 (2.5 to 3.4)

## Discussion

### Implications of the findings in context of existing research

Previous studies have demonstrated an association between socioeconomic variables (individuals’ SES, marital status and ethnicity) with morbidity and self-rated health. Low SES (low education [16, 30] and low annual household income [31, 32]) are associated with high morbidity and low self-rated health [33]. The same is true for marital status as single [34] and in Denmark, individuals of non-Western origin also have higher rates of morbidity [25]. There is no direct explanation as to why low SES, marital status as single and non-Western ethnicity are associated with high DOW. However, it could be speculated, whether the association between these socioeconomic variables and high DOW is caused by the general social gradient in health care both in detection as well as treatment of illness [35–37].

DOW was rated as high by 38.2% of the participants. This minority may be explained, as callers with life-threatening emergencies are prompted to contact the Copenhagen Emergency Medical Services and only the OOH services for less urgent situations. The majority of the participants (61.8%) reported a low DOW. This coincides with reports showing that approximately 50% of callers to OOH services are referred to selfcare or their GP the next day [38]. One could fear a possible risk of call handlers overlooking serious illness if patients report a low DOW. However, DOW is meant as an additional tool in the triage process by encouraging patients’ participation, increasing p*atient-centred communication* and thereby aiding call handlers in determining urgency. It should not stand alone. Furthermore, it is yet to be examined whether patients’ DOW when calling OOH services correlates to the DOW of a close relative/friend when calling on behalf of a patient. However, the same understanding applies with the use of DOW from a close relative/friend as from the patient herself, by encouraging caller participation, increasing caller-centred communication and giving callers the opportunity to voice their concerns.

### Strengths and limitations of this study

The main strength of this study is the use of data regarding SES, marital status and ethnicity obtained from registers, thereby minimizing information bias of self-report of socioeconomic variables. Furthermore, participants’ DOW was obtained when they were seeking help before being put through to a call handler, thereby limiting the influence of the triage result. Limitations include a possible risk of selection bias among callers who declined to participate, however, selection bias was assessed in a previous study [14] and no differences between participants and non-participants regarding gender, age, reason for contacting OOH services and triage response were found. Non-participation may, however, be more pronounced among callers with low SES due to low health literacy, callers with non-Western ethnicity due to possible language difficulties [39] and callers who were extremely distressed when contacting the OOH services. One may also argue that, as callers with low SES, marital status as single and non-Western ethnicity have higher morbidity and lower health literacy rates [19, 40], naturally they would more often have an increased sense of medical urgency, an association that previously been shown [41], leading to a high DOW. This possible bias, however, remains to be explored. The data includes missing variables of education and household income. The data on highest attained educational level is a little below the general coverage of the register (95.7% vs 96.4%) and within the category of missing information an association with high DOW is observed.

### Relevance of this study: possible implications for health care providers and policy makers

Previous studies have shown that the use of OOH services is significantly higher in areas with low SES [42] and it is therefore important to understand callers’ motivation for help-seeking in this context. As call handlers in OOH services cannot rely on visual cues in order to assess urgency during telephone triage, they must solely rely on verbal information given by callers, which may obscure their assessment of urgency. Moreover, as callers are unknown to the call handlers, their full medical history, SES and level of health literacy is not necessarily known. Experts within the field of emergency medicine decision-making recognise, that call handlers’ perceptions may be influenced by mental shortcuts and an unconscious integration of preconceptions and stereotyping [2, 43, 44]. The clinical relevance of this study does not lie in the statistical significance, but in the approach to the patient, and serves as a reminder that the symptoms the clinician deduce from a patient is the interpreted narrative. Incorporating DOW as an additional permanent triage tool may aid the triage process by encouraging patient participation, increasing patient-centred communication and giving callers the opportunity to voice their concerns, which may especially be advantageous to callers with lower health literacy or challenged verbal ability. Callers’ DOW could thereby, act as a mental forcing strategy, potentially debiasing the call handler’s perception of the caller. According to the Common-Sense Model of Self-Regulation (CSM) by Leventhal [45], a widely recognized theoretical framework, patients’ perceptions of their illness, is based on prior experience, personal beliefs, discussions with others and cultural understandings [46] and a relation between a patient’s illness representation and self-evaluation of urgency or DOW has previously been shown [12]. If the callers’ DOW, therefore, does not correspond to the call handler’s sense of urgency, the call handler may ask more in-depth questions, leading to a better understanding of the patient’s illness perception, especially in relation to DOW, possibly influencing and correcting the call handlers sense of urgency. Acknowledging the patient’s DOW as valuable triage information will enable elicitation, attention to, and understanding of the individual meaning and significance of patient’s symptoms [47].

Thus, when call handlers are presented with a high DOW from a caller, especially if this does not correspond to the call handler’s perception of urgency, an awareness of the significant association to various socioeconomic variables and high DOW, should urge call handlers to a more thorough questioning of the caller for possible severe illness, as this population group may have a higher risk of serious illness. This increase in patient-centred communication may aid the call handler in determining the correct type of health care needed, thus ensuring patient safety. Furthermore, the association of callers’ SES, marital status and ethnicity with DOW when contacting OOH services adds to the growing awareness of the socioeconomic inequality in health and healthcare and the continuing necessity of minimizing this gap. This is a new area of research and this study gives direction for future research to further strengthen the evidence.

## Conclusion

### Summary of main findings

There is a social and ethnic gradient in self-reported DOW when contacting an OOH, with low SES, marital status as single and non-Western ethnicity being associated with high DOW.

## Data Availability

The datasets used and/or analysed during the current study are available from the corresponding authors on reasonable request.

## Abbreviations

OOH: Out-of-hours
DOW: Degree-of-worry
SES: Socioeconomic status
GP: General practitioner
PIN: Personal identification number
ISCED: International Standard Classification of Education
DKK: Danish Krone
OR: Odds ratios
CI: Confidence intervals
SD: Standard deviation
